# Institutional violence and quality of service in obstetrics are associated with postpartum depression

**DOI:** 10.1590/S1518-8787.2017051006549

**Published:** 2017-07-07

**Authors:** Karina Junqueira de Souza, Daphne Rattner, Muriel Bauermann Gubert

**Affiliations:** I Unidade de Nutrição Clínica. Divisão de Apoio Diagnóstico e Terapêutico. Hospital Universitário de Brasília. Brasília, DF, Brasil; II Departamento de Saúde Coletiva. Faculdade de Ciências da Saúde. Universidade de Brasília. Brasília, DF, Brasil; III Departamento de Nutrição. Faculdade de Ciências da Saúde. Universidade de Brasília. Brasília, DF, Brasil

**Keywords:** Depression, Postpartum, epidemiology, Risk Factors, Violence, Quality of Health Care, Humanization of Assistance, Maternal-Child Health Services

## Abstract

**OBJECTIVE:**

To investigate the association between institutional violence in obstetrics and postpartum depression (PP depression) and the potential effect of race, age, and educational level in this outcome.

**METHODS:**

This is a cross-sectional study about the health care conditions for the maternal and child population of the Federal District, Brazil, carried out in 2011. The study has used a probabilistic sample of 432 women, whose children were aged up to three months, stratified by clusters. Indicators of institutional violence and demographic characteristics have been used in a logistic regression model to estimate the probability of occurrence of postpartum depression.

**RESULTS:**

The model has identified a high prevalence of postpartum depression, being it higher among non-white women and adolescent females, besides having a strong positive association between the several indicators of obstetric violence and postpartum depression. Positive interactions on a multiplicative scale have also been observed between: violence by negligence by health care professionals and race and age; physical violence from health care professionals and age; and, verbal violence from health care professionals and race.

**CONCLUSIONS:**

The indicators adopted to reflect institutional violence in obstetric care are positively associated with postpartum depression, which calls for a reflection on the need to make the health care protocols adequate to the precepts of the Brazilian humanization of childbirth care policies and changes in the obstetric care model.

## INTRODUCTION

Improving maternal health was the fifth goal for the millennium as defined by the Millennium Summit of the United Nations, in 2000, in New York. The objective of this event was to evaluate the leading world problems. Among the indicators proposed to measure the quality of health of pregnant women were: the increase of deliveries assisted by qualified health care professionals and a 75% decrease of the maternal mortality ratio, taking as baseline the value of this indicator for 1990. Some of these indicators in Brazil are close to meeting the goals stipulated by the World Health Organization (WHO); however, other indicators used to measure the quality of care at childbirth – such as those associated with gender issues – have not been privileged. In addition, despite the great advance that the Brazilian Unified Health System (SUS) represents to the country’s health care, there are still serious problems related to the access to reproductive health services and to the quality of the care offered for childbirth^20^. Among the problems related to the health of pregnant women, concerns have been raised more recently regarding certain practices adopted in medical assistance, referred to by specialists as “institutional violence in childbirth” or “obstetric violence”^5^ ^,^ ^7^ ^,^ ^9^ ^,^ ^10^ ^,^ ^12^ ^,^ ^13^ ^,^ ^21^ ^,^ ^24^.

According to the Brazilian Ministry of Health^19^, institutional violence is defined as the failure to act or any type of omission in health care services. This ranges from the broad level of lack of access to these services to their bad quality.

Some epidemiological studies have associated the occurrence of psychiatric disorders in the puerperal period, among them postpartum depression, with elements related to obstetrical care^2^ ^-^ ^4^ ^,^ ^11^ ^,^ ^16^ ^,^ ^23^ ^,^ ^2^ ^7^ ^,^ ^29^, such as feeling of abandonment during delivery, inadequate pain management, frustration for having delivered via cesarean section when natural childbirth was possible, and the pregnant woman’s perception of the team who provided the care. The negative impacts resulting from psychiatric disorders in the postpartum period are clearly harmful to the bonding between mother and child.

This study aimed to investigate the association between institutional violence in obstetrics and postpartum depression and the potential effect of race, age, and educational level in this outcome.

## METHODS

This study used the data from the research “Diagnosis of the health conditions and of the health care provided to the maternal and child population of the Federal District”, carried out during the national multi-vaccination campaign of 2011 in the Federal District (DF), Brazil, and it was approved by the Research Ethics Committee of Faculdade de Ciências da Saúde of the Universidade de Brasília (Process 13010).

The sampling strategy adopted was probabilistic, complex, by clusters, in two stages, and representative of the DF and for all administrative regions of the DF. The first stage consisted of choosing the health care centers, and the second stage, the mother-child pairs. The eligible health care centers were selected by randomization (maintaining representation for administrative regions). A total of 25 health care centers were selected, in 21 of the 30 administrative regions of the DF in 2010. The second stage referred to the systematic selection of mother and child pairs in the vaccination queue. In order to preserve the randomization criteria sample, each selected health center had a determined sampling interval, according to the estimate of attendance based on the number of children vaccinated at that unit in the campaign of the previous year (interval ranging from 1: 2 to 1:10 mothers/children).

This study used the part of the sample consisting of mothers whose children were aged up to three months and who answered the questions about postpartum depression; this estimate used a supposed prevalence of 20%. The calculated sample size was 432 women, with a 95% confidence interval and sampling error of 5%. The inclusion criteria were: mother with children younger than three months of age on the vaccination day.

The expansion of the sample for the population of the DF was calculated by the sample weight of each individual, respecting the representativeness of each sampling unit in the calculation of the sample. For this estimate, the inverse of the probability for each health center selected was multiplied by the number of individuals who effectively took part in the campaign, under the total number of cases collected. After the expansion, the sample represented a universe of 10,468 women.

For data collection, a previously tested form was used. Mothers were questioned about care for delivery, type of delivery, referral or not to a maternity hospital or need to search for a hospital bed, presence of a companion before, during, and immediately after childbirth, if they reported any violence in the relationships with health care professionals, and postpartum depression, besides information on family characteristics. Information about race or color perception was collected (initially in four categories: white, black, yellow, and indigenous) and aggregated into two categories (white and others – non-white). The education of the mother was measured asking the mother about the last completed year of education, and this information was aggregated into two categories of educational level: elementary or middle school (equal or less than 12 years) or university (more than 12 years at schooling). The age of the mother was calculated using the given birth date, and aggregated into two categories: less than 20 years old (adolescents) and 20 years old or more (adults).

The shorter version of the Edinburgh Postnatal Depression Scale (EPDS-6) was adopted for screening of postpartum depression and it was validated by Malloy-Diniz et al.^15^ in 2010. The best cutoff point for screening of depression symptoms suggested by the authors is the score of ≥ 6 (81% sensibility; 86% specificity). Santos^26^ has compared the complete version of the Edinburgh scale with the shorter version and found an almost-perfect agreement between both scales. Postpartum depression is the dependent variable in this study.

The conceptual model of quality of care at childbirth used to build the indicators of violence is based on a thesis that interactions between women, health care professionals, health care facilities, and society can influence the care at childbirth, and violence can be present in those relations^25^.

To build the indicators for the analysis, “zero” meant the answer “no” and “one” meant the answer “yes” to these questions. The indicator for violence in the relationship between the parturient and the health care system – VSis – was defined by the answer yes to any of these questions: a) Did you go to more than one hospital to find a bed for childbirth? and b) Did your delivery not happen in the hospital originally recommended? The indicator to measure institutional violence in the relationship between the parturient and the health care services – VSer – was defined by the answer yes to the questions: a) Were you not allowed a companion of your own choice during labor?; b) during childbirth?; and, c) after childbirth? The indicator to measure violence in the relationship of the parturient with the health care professionals – VP – considered three types: those related to physical violence – VPF –, those related to verbal violence – VPV –, and those related to violence by negligence – VPN. Physical violence was defined as the answer yes to the questions: During childbirth, did any of the health care professionals: a) hurt you during the vaginal exam?; b) hit you?; c) push you?; and, d) tie you? Verbal violence was defined by the answer yes to any of the questions: During delivery, did any of the health professionals: a) yell at you?; or said something similar to b) Don’t cry! Next year you will be here again; c) When you were making the baby, you didn’t cry, or called your mommy. Why are you crying now?; d) If you keep screaming, I will stop what I’m doing and I won’t come back again; and, e) If you keep screaming, you will harm your baby. It will be born deaf. Violence by negligence was defined as the answer yes to the questions: a) Did the health care professionals deny you pain relief?; b) Did any health care professional deny assistance?; and, c) Did any health care professional not explain the procedures he or she was doing to you? These indicators are the independent variables in the study.

The descriptive analysis for sample characterization adopted central tendency and dispersion and frequency measures. Bivariate analysis, by Pearson’s Chi-square test, was used to estimate the association of postpartum depression with the categories of institutional violence and with the categories of demographic and socioeconomic variables. Prevalence of postpartum depression for each variable and its 95% confidence intervals was calculated. Univariate logistic regression was used to calculate the crude odds ratio. For multivariate analysis, a logistic regression model was built including all the variables of interest, in which postpartum depression is the dependent variable and the other ones related to violence are the independent variables. Race, color, and educational level were used as control confounding variables. To access interaction, the product of each two evaluated terms was added to the model, which were: race and VPN, race and VPF, race and VPV, age and VPN, age and VPF, age and VPV, schooling and VPN, schooling and VPF, and schooling and VPV. The modeling strategy adopted to estimate the adjusted odds ratio was the backward stepwise method. The model begins with all predictors included. The computer then tests whether any of those predictors can be removed from the model without having a substantial effect on how well the model fits the observed data. The criterion to include variables in the model was their association with the occurrence of postpartum depression – p < 0.20 in the bivariate analysis (Pearson) – and a theoretical relevance for the analysis. By this criterion, we included race, age, and educational level in the model. For the final model, we kept the variables that continued to present an association with the outcome when adjusted by all other variables (p < 0.05 in the Wald test) and those that were considered relevant by the literature, besides those that composed the interactions, when these were significant.

## RESULTS

The total sample, after expansion, comprised 10,468 women. The average age of women with children under three months of age, included in this study, was 28.8 years (14–45 years, SD = 7.24 years), of which 59.9% were non-white. Regarding schooling, 42.6% of the women had complete or incomplete university education. Table 1 describes their demographic and socioeconomic characteristics.

**Table 1:** Study population according to demographic, socioeconomic, and obstetric care variables. Federal District, Brazil, 2011. (n = 10,468)

Variable	Average	Standard deviation
		
Number of prenatal care visits	9.6	3.6
	n	%
Age (years)		
< 20	958	9.2
≥ 20	9,510	90.8
Race		
White	4,198	40.1
Non-white	6,270	59.9
Schooling		
Elementary/Middle school	6,010	57.4
University	4,458	42.6
Registered in government programs^a^		
Yes	1,612	15.4
No	8,856	84.6
Delivery at the referred hospital		
Yes	6,658	63.6
No	3,810	36.4
Type of delivery^b^		
Vaginal	2,774	26.5
Cesarean section	7,558	72.2
Skin-to-skin contact or breastfeeding during 1st hour		
Yes	8,237	78.7
No	2,098	20.0
Companion during delivery^b^		
Yes	5,795	55.4
No	4,540	43.4
Rooming-in^b^		
Yes	9,489	90.7
No	846	8.1
Puerperal or child welfare appointment scheduled^b^		
Yes	8,650	82.6
No	1,686	16.2

^a^ Recipients of a monthly allowance.

^b^ 1.2% of non-response.

For obstetric care, 97.8% of the respondents said they had prenatal care. For 37.1% of the women, the first visit was in the first month of pregnancy, and for 35.3% it was in the second month.

The public hospitals of the DF were responsible for 49.1% of the deliveries, and the overall proportion of cesarean sections was 72.2%. Women classified the quality of prenatal care as very good (45.6%) and good (39.7%).

Regarding the setting of delivery, 64.1% of the women were referred to hospitals during prenatal care, although only 53.3% of them did, in fact, have their delivery at the previously referred hospital. For 33.7% of them, there was no hospital referral at all. The number of hospitals sought for delivery was 1.57 (1–11, SD = 0.91) on average.

For institutional routines for care during and immediately after delivery, only 55.1% of the women reported they had a companion of their own choice during delivery, and 25.6% explained they were not allowed by the services. Skin-to-skin contact between mother and child, or the child being breastfeed during the first hour of life, was reported by 78.7% of them women, whereas rooming-in was available for 91.8% of them. Only 2.6% of the women considered the quality of care at delivery bad or very bad; however, there were complaints regarding painful vaginal exams (6.9%), inadequate pain relief (6%), poor service (2.4%), and lack of communication and explanations of the professional as to the obstetric procedures being performed (3.6%). Use of offensive language by health care professionals during childbirth care was also reported. Table 1 also summarizes the characterization of the studied population according to some obstetric care variables.

The prevalence of postpartum depression in the studied population was 18.4%. This prevalence in women with children aged up to 3 months was higher among non-white women (prevalence ratio = 1.7), those who had finished middle school (prevalence ratio = 1.6), and those who were aged under 20 years (prevalence ratio = 2.8). Table 2 describes the prevalence of postpartum depression with its respective confidence intervals for some selected variables.

**Table 2:** Prevalence of postpartum (PP) depression and 95% confidence intervals (95%CI) in women with children under three months of age. Federal District, Brazil, 2011.

Variable	Prevalence of postpartum depression (%)	95%CI	p*
Age (years)			< 0.00
< 20	43.3	40.1–46.5	
≥ 20	15.4	14.6–16.3	
Race			< 0.00
Non-white	21.5	20.5–22.6	
White	12.4	11.2–13.7	
Schooling			< 0.00
Elementary/Middle school	21.3	20.2–22.4	
University	12.9	11.6–14.2	
Place of delivery			< 0.96
SUS hospital	18.5	17.4–19.6	
Non-SUS hospital	18.5	17.2–19.8	
Companion during delivery			< 0.00
Yes	11.9	10.9–12.9	
No	25.7	24.3–29.0	
Demanded more than 1 hospital			< 0.50
Yes	18.9	17.5–20.3	
No	18.3	17.3–19.3	
Physical violence			< 0.00
Yes	59.0	54.7–63.1	
No	15.8	15.0–16.6	
Violence by negligence			< 0.00
Yes	51.8	48.0–55.5	
No	15.5	14.7–16.4	
Verbal violence			< 0.00
Yes	50.3	47.0–53.5	
No	14.5	13.7–15.4	
Violence from the institution			< 0.00
Yes	26.1	24.8–27.6	
No	12.2	11.3–13.2	
Violence from the health system			< 0.00
Yes	19.6	18.6–20.6	
No	16.1	14.7–17.6	

SUS: Brazilian Unified Health System

* Pearson’s Chi-square test.

Table 3 presents the crude odds ratio and its respective confidence intervals for the association between the outcome and the variables related to violence in obstetric care. All the selected variables, when analyzed individually, were strongly associated with a higher risk of postpartum depression. Women who suffered violence by negligence at childbirth had a seven times higher risk of developing postpartum depression than women who did not. Both physical and verbal violence were also strongly associated with postpartum depression (5.83 and 5.93, respectively). It is noteworthy that the presence of a companion during delivery was a protective factor against postpartum depression.

**Table 3:** Crude odds ratio (OR) for postpartum depression (and 95%CI) in women with children under three months of age, according to selected variables. Federal District, Brazil, 2011.

Variable	Crude OR	95%CI
Violence from the system	1.27	1.11–1.43
Violence in the service	2.54	2.31–2.80
Violence by negligence by HP	7.66	6.37–9.23
Physical violence by HP	5.83	4.95–6.87
Verbal violence by HP	5.93	5.14–6.87
Delivery at SUS hospital	1.00	0.90–1.10
Companion during delivery	0.39	0.30–0.34
Demanded more than 1 hospital	1.03	0.92–1.16

HP: health care professionals; SUS: Brazilian Unified Health System

The logistic regression model described in Table 4 shows that physical violence (OR = 1.51; 95%CI 1.1–2), in the relationship between the parturient and health care professionals, is the most important determinant of postpartum depression, more than the violence related to the health care system (OR = 0.84; 95%CI 0.7–0.9) and to the health services (OR = 1.34; 95%CI 1.2–1.6). In addition, the model suggests that race and age are variables that modify the effect of all violence in the relationship with health professionals: physical, verbal, and by negligence.

**Table 4:** Logistic regression model with adjusted odds ratio (OR) for postpartum depression in women with children under three months of age, according to selected variables. Federal District, Brazil, 2011.

Variable	β	Standard error	OR	95%CI	p^a^
Intercept	-2.18	0.08	0.11	-	0.00
Non-white race	0.16	0.08	1.17	1.01–1.37	0.04
Age < 20 years	1.11	0.09	3.02	2.49–3.66	0.00
Violence from the system	-0.17	0.07	0.84	0.73–0.97	0.02
Violence from services	0.03	0.07	1.34	1.16–1.56	0.00
Violence by negligence by HP	-0.81	0.43	0.44	0.19–1.04	0.06
Physical violence by HP	0.41	0.15	1.51	1.13–2.02	0.00
Verbal violence by HP	0.24	0.29	1.28	0.71–2.29	0.41
IVP by negligence * race	2.82	0.46	16.76	6.87–40.89	0.00
IVP by negligence * age	-2.30	0.28	0.10	0.03–0.15	0.00
IVP physical violence * age	1.76	0.27	5.81	3.39–9.98	0.00
IVP verbal violence * race	1.27	0.32	3.54	1.91–6.59	0.00

HP: health professionals; IVP: interaction of violence by health professionals

^a^ Wald test (multiple logistic regression).

The odds ratio for the product of violence by negligence from health care professionals and race shows a positive interaction on a multiplicative scale, OR = 16.76 (95%CI 6.8–40.9). Considering the same control variables, race increases in 1.17 times the risk of developing depression in individuals who did not suffer violence by negligence, whereas, in those who did suffer it, the risk of developing depression is 19.69 times higher [Exp(0.16+2.82)].

As for the age effect, once exposed to physical violence by a health care professional, the effect of younger age increased in approximately 17 times the risk of the woman developing depression [Exp(1.11+1.76)]. However, individuals who showed the same values for the control variables and who were not subjected to violence by negligence had a risk of developing depression three times higher than those who did suffer this type of violence. Moreover, younger individuals who suffered violence by negligence had a lower risk of developing depression, in the order of 0.3 times [Exp(1.11-2.3)].

There was also an interaction between verbal violence by a health care professional and race, being it a positive interaction on a multiplicative scale, with an OR = 3.54 (95%CI 1.9–6.6). Keeping the values for the control variables, the effect of race on individuals subjected to verbal violence increased in approximately four times the risk of postpartum depression [Exp(0.16+1.27)].

## DISCUSSION

Psychiatric disorders during the puerperal period affect a significant number of women and can negatively affect the health of mother and baby^3^ ^,^ ^6^ ^,^ ^27^ ^,^ ^28^. This study identified a high prevalence of postpartum depression in non-white adolescent women, besides a strong positive association between the several indicators of violence in obstetric care and postpartum depression. Some other studies have found a higher prevalence of postpartum depression, such as the prospective cohort study by Brito et al.^6^ and Ludermir et al.^14^ The differences observed might be due to study design. Moreover, selecting women who bring their children to vaccination will, probably, underestimate the prevalence of postpartum depression, as women with postpartum depression are more unlikely to bring their children to vaccination.

Positive interactions on the multiplicative scale were observed between violence by negligence by a health care professional and race and age, physical violence by a health care professional and age, and verbal violence by a health care professional and race.

Unfortunately, the broad spectrum of psychiatric disorders related to the puerperal period are poorly understood and, consequently, under-diagnosed and under-treated^16^. Research in the neurobiology area suggests that highly stressful experiences of violence and threats can lead to a hyper-activation and deregulation of the autonomous nervous system, translating stress into emotional disorders. Although postpartum depression has a multifactorial origin, researchers on mental health agree that trauma is a risk factor for the development of depression and other psychiatric disorders^3^ ^,^ ^27^ ^,^ ^29^.

Menage^18^, while studying the association of psychological stress caused by obstetric and gynecological procedures, has found a high correlation between variables related to childbirth care and psychiatric disorders, such as feeling of loss of control by the woman, lack of information given by the health care professional, experience of physical pain, perception of not having been adequately taken care of by the health care team, and submission to procedures without informed consent. In critical reviews of the literature, Olde et al.^23^ and Zambaldi et al.^29^ have observed that inadequate pain relief, feeling of loss of control by the woman, a humiliating experience, lack of information and support by the health care team, and fear of own or baby’s death were risk factors for the development of psychiatric disorders. Several studies have shown a positive association between events related to delivery and postpartum depression. Alvarado-Esquivel et al.^2^ have found odds ratio of 7.71 for women with postpartum depression subjected to stress during delivery. A study on the subjective configurations of postpartum depression^27^ has reported that some experiences are associated with the onset of a postpartum depression episode, such as those related to the feeling of abandonment, inadequate pain control, and feeling of frustration of women who were subjected to a cesarean section when natural childbirth was possible. In another research^27^, 60% of the women who considered their delivery traumatic showed depression symptoms in the first six weeks after childbirth. The indicators used in our study to measure institutional violence in obstetric care strengthen these results.

The indicator of violence related to the health care system, represented by the search for a hospital bed during labor, showed significant positive association with postpartum depression in the univariate analysis; however, it was not a risk factor for the development of postpartum depression when other variables were included in the model. The access to reproductive health care services, the search for health care assistance, and the long waits are characteristic of the Brazilian health care system and, because they are intrinsic to the culture, many times they are not considered a type of violence. Physicians, administrators, employees of the institutions, and the patients themselves accept that patients need to wait to receive assistance^7^ ^,^ ^8^. It contradicts the WHO recommendations and Brazilian Law 11,634/2007 that a parturient has a guaranteed right to know in which maternity she will deliver. The search for care puts the mother’s and baby’s health at risk; and black, indigenous, and migrant women are the ones who suffer more discrimination in their access to health care services and who are usually the most vulnerable to this type of institutional violence. According to Deslandes^8^, these failures of the health care system are important barriers to the development of humanized care during childbirth and evidence the disconnection of the health care network for women.

The indicator of institutional violence in health care services, after adjusted for other variables, was significantly associated with the outcome. Suffering violence in health care services increases in approximately six times the probability of women developing postpartum depression; the univariate analysis has already shown the protective role of the presence of a companion. Even though Brazilian Law 11,108, from 2005, guarantees the right of a companion of the woman’s own choice before, during, and immediately after delivery in the hospitals of the Brazilian National Unified Health System (SUS), and by-law GM 2418/2005 guarantees this same right to women cared at private hospitals under contract with the SUS, this was not a reality reported by the women interviewed in our study.

However, despite being determinant of postpartum depression in the univariate analysis, in the logistic regression, the violence related to the system and to health care services lost strength of association, evidencing the importance of violence by health professionals in the determination of postpartum depression. The indicators of violence in the relationship of the parturient with health care professionals showed the strongest positive association with postpartum depression. The term used to represent the interaction of the variable violence by negligence by a health care professional with the non-white race showed a positive coefficient, suggesting an increase of the effect on a multiplicative scale. The increase in the probability of developing postpartum depression, given the interaction between these two variables, can still be higher than that observed in the term that represents the interaction, if the values of each of the variables are considered individually. According to Diniz^10^, unnecessary surgeries, loneliness, and abandonment, which are “rooted” in routine procedures, are the social inequalities linked to gender, class, and race that increase discrimination even more. For Mattar et al.^17^, there are reproductive hierarchies, meaning that some maternities are more prestigious and respected than others, whereas others can be sources of prejudice, discrimination, and human rights violation.

A study done in 2008, in a large city of Brazil^1^ found that health care professionals recognize the use of discriminatory and disrespectful practices in the day-to-day care, such as pejorative language, threats, reprimands, and negligence in pain management. A research carried out in 2010, representative for Brazil in which 2,365 women from 25 states from both urban and rural areas were interviewed, found that one in each four women suffered some type of violence during delivery, especially a painful vaginal exam^a^. Data from another research on institutional violence in public maternities from the city of São Paulo has shown that pregnant women and parturients recognize discriminatory practices and rudeness in the care provided by health professionals, and these experiences are frequent, thus revealing the trivialization of this type of violence^10^.

In our study, despite the strong association observed between violence in obstetric care and postpartum depression, we should be careful when assuming causality based on the cross-sectional nature of the study, in which the outcome variable and the exposure to violence are investigated simultaneously. Moreover, the measure of association used, that is, the odds ratio, may overestimate the risk in conditions in which the prevalence of the outcome is higher. Other important potential limitation of the study is the absence of screening for depression prior to pregnancy, which, according to the consulted literature, is one of the main predictors of postpartum depression^3^. Another limitation of the study is the potential bias of a high educational level of the respondents – 42.6% of the women had been a student at some university – and it might had influenced the high number of prenatal care visits. As limitations of this study we can also include: non-qualification of received refusals and the possibility of mothers with postpartum depression not watching their children during the vaccination campaign, as a consequence of their depression. However, the prevalence of postpartum depression in this study (18.4%) is similar to that observed in the study “*Chamada Neonatal na Amazônia Legal e Nordeste”* from 2010, which was 18.0%^20^.

From the results of this research, we conclude that the indicators used to reflect institutional violence in obstetric care were positively associated with postpartum depression in the Brazilian Federal District. These results indicate the need of both the adequacy of obstetric care protocols and the sensitizing of health professionals to the need to change their attitudes and practices followed by changes in the obstetric care model.

## Footnotes

a Fundação Perseu Abramo. Mulheres brasileiras e gênero nos Espaços públicos e privados. São Paulo: SESC; 2010 [cited 2012 Nov 18]. Available from: http:/wwwfpabramo.org.br/sites/default/files/pesquisaintegra.pdf

## References

[1] Aguiar JM, D’Oliveira AFPL (2011). Institutional violence in public maternity hospitals: the women’s view. Interface.

[2] Alvarado-Esquivel C, Sifuentes-Alvarez A, Estrada-Martínez S, Salas-Martínez C, Hernándes-Alvarado AB, Ortiz-Rocha SG (2010). Prevalence of postnatal depression in women attending public hospitals in Durango, Mexico. Gac Med Mex.

[3] Anniverno R, Bramante A, Mencacci, Durbano FC, Durbano F (2013). Anxiety disorders in pregnancy and the postpartum period. New iInsights into anxiety disorders.

[4] Arrais AR (2005). As configurações subjetivas da depressão pós-parto: para além da padronização patologizante.

[5] Bowser D, Hill K (2010). Exploring evidence for disrespect and abuse in facility-based childbirth: report of a landscape analysis.

[6] Brito CNO, Alves SV, Ludermir AB, Araújo TVB (2015). Postpartum depression among women with unintended pregnancy. Rev Saude Publica.

[7] D’Oliveira AFPL, Diniz SG, Schraiber LB (2002). Violence against women in health-care institutions: an emerging problem. Lancet.

[8] Deslandes SF (2005). Humanization of care in maternity hospitals in Rio de Janeiro from the administrator’s perspective. Cienc Saude Coletiva.

[9] Dias MAB, Deslandes SF (2006). Patients’ expectations concerning childbirth care at a public maternity hospital in Rio de Janeiro, Brazil: challenges for the humanization of obstetric care. Cad Saude Publica.

[10] Diniz SG, Chacham AS (2006). O “corte por cima” e o “corte por baixo”: o abuso de cesáreas e episiotomias em São Paulo. Quest Saude Reprod.

[11] Garthus-Niegel S, Soest T, Vollrath ME, Eberhard-Gran M (2013). The impact of subjective birth experiences on post-traumatic stress symptoms: a longitudinal study. Arch Womens Ment Health.

[12] Gomes AMA, Nations MK, Luz MT (2008). “Stepped-on like a floor-mat”: human experience of hospital violence in the Northeast of Brazil. Saude Soc.

[13] Hotimsky SN, Rattner D, Venancio SI, Bogus CM, Miranda MM (2002). Childbirth as I see it... or the way I wish it was? Expectations of pregnant women towards childbirth and obstetric care in the public health care system. Cad Saude Publica.

[14] Ludermir AB, Lewis G, Valongueiro SA, Araújo TVB, Araya R (2010). Violence against women by their intimate partner during pregnancy and postnatal depression: a prospective cohort study. Lancet.

[15] Malloy-Diniz LF, Schlottfeldt CGMF, Figueira P, Neves FS, Correa H (2010). Edimburg Postpartum Depression Scale: factorial analyses and development of six items version. Rev Bras Psiquiatr.

[16] Marrs CR, Durette RT, Ferraro DP, Cross CL (2009). Dimensions of postpartum psychiatric distress: preliminary evidence for broadening clinical scope. J Affect Disord.

[17] Mattar LD, Diniz CSG (2012). Reproductive hierarchies: motherhood and inequalities in women’s exercising of human rights. Interface.

[18] Menage J (1993). Post-traumatic stress disorder in women who have undergone obstetric and/or gynaecological procedures: a consecutive series of 30 cases of PTSD. J Reprod Infant Psychol.

[19] Ministério da Saúde (BR), Secretaria de Políticas de Saúde (2002). Violência intrafamiliar: orientações para prática em serviço.

[20] Ministério da Saude (BR), Secretarira de Ciência, Tecnologia e Insumos estratégicos, Departamento de Ciência e Tecnologia (2013). Avaliação da atenção ao pré-natal, ao parto e aos menores de um ano na Amazônia Legal e no Nordeste, Brasil, 2010.

[21] Misago C, Kendall C, Freitas P, Haneda K, Silveira D, Onuki D (2001). From ‘culture of dehumanization of childbirth’ to ‘childbirth as a transformative experience’: changes in five municipalities in north-east Brazil. Int J Gynaecol Obstet.

[22] Morse ML, Fonseca SC, Gottgtroy CL, Waldmann CS, Gueller E (2011). Severe maternal morbidity and near misses in a regional reference hospital. Rev Bras Epidemiol.

[23] Olde E, Hart O, Kleber R, Son M (2006). Posttraumatic stress following childbirth: a review. Clin Psychol Rev.

[24] Rattner D (2010). Humanizing childbirth care: a brief theoretical framework. Rev Tempus Acta Saude Coletiva.

[25] Rattner D (2001). Quality of care at childbirth: seeking a comprehensive approach.

[26] Santos W (2013). A depressão pós-parto influencia o cuidado à saúde infantil?.

[27] Schwab W, Marth C, Bergant AM (2012). Post-traumatic Stress Disorder Post Partum: the Impact of birth on the prevalence of Post-traumatic Stress Disorder (PTSD) in multiparous women. Geburtshilfe Frauenheilkd.

[28] White T, Matthey S, Boyd K, Barnett B (2006). Postnatal depression and post-traumatic stress after childbirth: prevalence, course and co-occurrence. J Reprod Infant Psychol.

[29] Zambaldi CF, Cantilino A, Montenegro AC, Paes JA, Albuquerque TLC, Sougey EB (2009). Postpartum obsessive-compulsive disorder: prevalence and clinical characteristics. Compr Psychiatry.

